# Thermosensitive Hydrogel Enables Noninvasive Extracellular Vesicle Therapy for Atopic Dermatitis

**DOI:** 10.34133/bmr.0368

**Published:** 2026-05-18

**Authors:** Doil Park, Mingyu Jeon, Jooho Kim, Ji Eun Kim, Yeonjeong Kim, Kyung Min Park, Eun Hee Kim, Jae Min Cha, Oh Young Bang

**Affiliations:** ^1^ Department of Health Sciences and Technology, Samsung Advanced Institute for Health Sciences and Technology (SAIHST), Sungkyunkwan University, Seoul 06355, Republic of Korea.; ^2^ Department of Biomedical & Robotics Engineering, College of Engineering, Incheon National University, Incheon 22012, Republic of Korea.; ^3^ S&E bio, Inc., Seoul 06351, Republic of Korea.; ^4^ Department of Bioengineering and Nano-Bioengineering, College of Life Sciences and Bioengineering, Incheon National University, Incheon 22012, Republic of Korea.; ^5^ Research Center for Bio Materials & Process Development, Incheon National University, Incheon 22012, Republic of Korea.; ^6^ Department of Neurology, Samsung Medical Center, Sungkyunkwan University School of Medicine, Seoul 06351, Republic of Korea.

## Abstract

Atopic dermatitis (AD) is a chronic inflammatory skin disease characterized by impaired barrier function and helper T cell 2 (Th2)-dominant immune dysregulation. Subcutaneous administration of mesenchymal-stem-cell-derived extracellular vesicles (MSC-EVs) has shown substantial efficacy in preclinical AD models; however, its clinical utility remains restricted by injection-associated pain and poor patient compliance, necessitating more patient-friendly delivery strategies. Here, we propose a patient-friendly, noninvasive therapeutic strategy using clinical-grade MSC-EVs incorporated into a thermosensitive hyaluronic acid/Pluronic F127 (HP) hydrogel. The optimized HP hydrogel exhibited desirable shear-thinning behavior and tunable sol–gel transition at body temperature, enabling sustained EV release. In a *Dermatophagoides farinae*-extract-induced AD mouse model, topical application of HP-loaded EVs (HP+EVs) suppressed AD symptoms, epidermal thickening, and mast cell infiltration, demonstrating an efficacy comparable to that of subcutaneous EV injections. Notably, HP+EV treatment down-regulated both Th2 and Th1/pro-inflammatory cytokines, restoring their levels to those in healthy controls. In addition, EVs rapidly penetrated the disrupted skin barrier in AD lesions via surface-protein-dependent mechanisms, as evidenced by the loss of permeability following proteinase K treatment. Furthermore, HP+EV treatment up-regulated barrier proteins and markedly down-regulated oxidative stress markers. This study demonstrates a major advancement in EV-based therapeutics, providing a promising noninvasive formulation for the treatment of AD.

## Introduction

Atopic dermatitis (AD) is a chronic inflammatory skin disease characterized by skin barrier disruption and immune dysregulation, often triggered by genetic susceptibility and environmental factors such as house dust mites [1]. The clinical efficacy of existing AD treatments is often constrained by side effects associated with their delivery methods as well as their diverse pharmacological mechanisms. Biological agents such as dupilumab, which function by inhibiting helper T cell 2 (Th2)-mediated immune response, have demonstrated substantial efficacy in the management of moderate-to-severe AD [2]. However, these treatments rely on repeated subcutaneous injections that are associated with pain, “needle phobia”, and poor treatment adherence, particularly in the pediatric population, which comprises a large proportion of patients with AD. In contrast, conventional topical treatments, including corticosteroids and calcineurin inhibitors, are noninvasive but are often associated with local side effects (e.g., skin atrophy and burning sensations) and safety concerns regarding long-term use [3]. Moreover, the therapeutic efficacy of such existing topical formulations is frequently limited by the robust barrier function of the stratum corneum, which restricts the penetration of macromolecular drugs and leads to poor therapeutic outcomes in severe cases [4]. Therefore, there remains a critical unmet clinical need for a novel therapeutic platform that integrates the high efficacy of biological agents with the safety and convenience of topical administration while simultaneously mitigating potential side effects and overcoming skin barrier permeation challenges.

Previously, we demonstrated that clinical-grade mesenchymal-stem-cell-derived extracellular vesicles (MSC-EVs; SNE-101) exert potent therapeutic effects in animal models of AD. In these studies, EVs were administered via direct subcutaneous injection into skin lesions to effectively suppress inflammation and modulate immune responses [5,6]. Despite the promising results in in vivo efficacy, the invasive nature of injection-based delivery remains a hurdle for its widespread clinical adoption. Although the topical application of EVs could ideally resolve this issue, simple liquid formulations of EVs typically suffer from rapid clearance, desiccation, and loss of bioactivity at the lesion site [7,8]. Furthermore, because the compromised epidermal barrier in AD is extremely vulnerable to mechanical stress [9], the delivery vehicle must prevent physical friction during application to avoid exacerbating inflammation. Consequently, there is a persuasive need for an advanced delivery vehicle capable of overcoming these challenges.

**Fig. 1.**
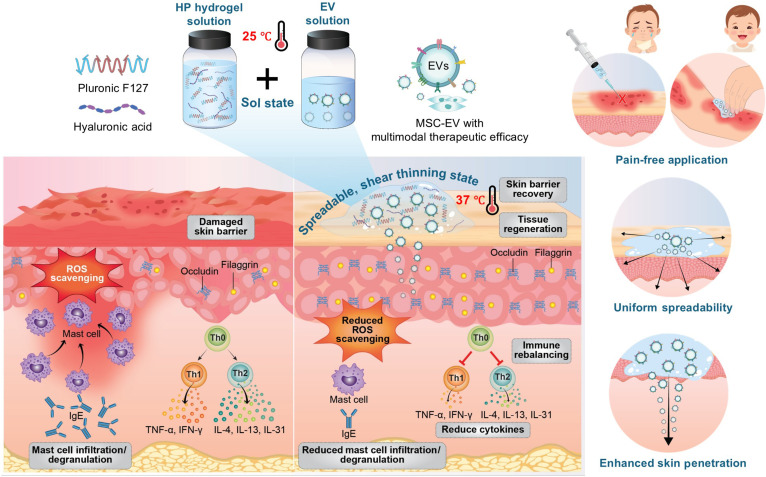
Schematic illustration of the thermosensitive hydrogel-mediated topical EV delivery platform for atopic dermatitis (AD) therapy. HP, hyaluronic acid/Pluronic F127; EV, extracellular vesicle; MSC-EV, mesenchymal stem cell extracellular vesicle; ROS, reactive oxygen species; TNF-α, tumor necrosis factor-alpha; IFN-γ, interferon-gamma; IgE, immunoglobulin E; IL, interleukin; Th, helper T cell.

To develop an ideal topical vehicle for AD, selecting an appropriate hydrogel is critical, as conventional gelation processes can easily compromise EV stability. For instance, photo-cross-linking methods (e.g., gelatin methacryloyl) generate reactive oxygen species (ROS) that damage EV lipid membranes and internal cargos [10]. Additionally, natural physical hydrogels like chitosan inherently require acidic solvents for dissolution [11], leading to altered surface charges and irreversible EV aggregation [12]. To strictly preserve EV bioactivity while ensuring patient compliance, we developed a thermosensitive hydrogel platform using an optimized combination of hyaluronic acid (HA) and Pluronic F127 (PF127). PF127 is a Food and Drug Administration (FDA)-approved polymer responsible for a unique sol–gel transition property, which enables our system to form a gel solely driven by body temperature at a physiological pH 7.4. This property allows the formulation to be applied as a liquid and immediately form a gel upon skin contact. This transition facilitates the homogeneous dissolution of drugs within the vehicle and ensures a prolonged residence time on the skin [13,14]. Importantly, the precise rheological tuning of this HA/PF127 (HP) formulation endows it with optimal shear-thinning properties, guaranteeing friction-free spreadability on sensitive AD lesions. HA further contributes by creating a hydrated microenvironment that facilitates tissue repair and potentially aids in transepidermal delivery of nanoparticles [15]. By encapsulating EVs within this optimized HP system, we sought to create a noninvasive delivery strategy that eliminates injection-related pain while providing sustained EV release and penetration [16]. Crucially, unlike conventional topical corticosteroids or calcineurin inhibitors that merely suppress inflammation at the cost of inducing skin atrophy over long-term use [17], this EV–hydrogel platform provides a targeted biologic therapy. It not only rebalances the immune microenvironment but also actively promotes tissue regeneration and barrier restoration, offering a fundamentally safer and sustainable alternative for chronic AD management. The therapeutic efficacy of this HP-loaded EV (HP+EV) formulation was validated in a *Dermatophagoides farinae* extract (DFE)-induced AD mouse model. Our comparative analysis with direct EV injections demonstrates that topical HP+EV application effectively modulates Th1/Th2 immune responses, restores skin barrier integrity, and alleviates oxidative stress, thereby establishing this platform as a promising next-generation, patient-friendly therapy for AD (Fig. 1).

## Materials and Methods

All experimental procedures were conducted in accordance with previously validated standard operating procedures. The use of human-derived materials in this study was approved by the Institutional Review Board (IRB) of Samsung Medical Center. Wharton’s jelly (WJ) tissue was collected from 3 healthy donors, whose legal guardians provided written informed consent prior to participation (IRB No. 2016-07-102). This study adhered to the US FDA regulatory standards for human cells, tissues, and cellular/tissue-based products, specifically those defined under Title 21 CFR Part 1271, Subpart 361 of the Public Health Service Act. All animal experiments were reviewed and authorized by the Institutional Animal Care and Use Committee at the Samsung Medical Center’s Laboratory Animal Research Center, an Association for Assessment and Accreditation of Laboratory Animal Care International-accredited facility. Animal handling and reporting conformed to the Animal Research: Reporting of In Vivo Experiments guidelines for in vivo studies [18].

### Preparation of 3D spheroid cultures of WJ-MSCs

Human WJ-derived mesenchymal stem cells (WJ-MSCs) were cultured in a growth medium in a 5% CO_2_ incubator at 37 °C until passage 5. Passage 6 cells were used to establish 3-dimensional (3D) spheroid cultures. The WJ-MSCs were seeded onto microwell-patterned plates, rinsed with phosphate-buffered saline (PBS), and dissociated using TrypLE Express (Gibco, Grand Island, NY, USA). After centrifugation, the cell pellet was resuspended in protein- and serum-free medium, and cell numbers were determined using a hemocytometer.

A 60-ml aliquot of this suspension was loaded into a microarray plate containing approximately 69,000 microwells (each 500 μm in diameter and 200 μm in depth), precoated with 2-methacryloyloxyethyl phosphorylcholine polymer at a seeding density of 400 cells per well. For 3D spheroid formation, cells were cultured statically in α-minimal essential medium without antibiotics for 4 d at 37 °C in a humidified CO_2_ incubator, allowing spontaneous aggregation into spheroidal cell clusters.

### Isolation of EVs

The culture supernatants were gently collected from the top of each well using pipettes. To eliminate cellular debris and apoptotic bodies, 1,800 ml of conditioned medium underwent centrifugation at 2,500 × *g* for 10 min, followed by membrane filtration through a 0.22-μm pore-size filter. The clarified medium was then subjected to tangential flow filtration using a 300-kDa molecular weight cutoff (MWCO) modified polyethersulfone hollow fiber filter module (MiniKros, Spectrum Laboratories, Rancho Dominguez, CA, USA) installed on a KrosFlo KR2i system and optimized for large-scale EV processing. During filtration, EVs were retained in the circulation line (retentate), while smaller molecules, such as free proteins, passed through the membrane and were collected as permeates. The permeate fraction is defined as the secretome. The EV-rich retentate was then concentrated in a sterile collection bag. Five sequential buffer exchanges were performed with PBS, and the EV solution was concentrated to a final volume of 300 ml in PBS. A final 0.22-μm filtration step was carried out before downstream applications.

All isolation procedures were conducted in compliance with the Korean Ministry of Food and Drug Safety (MFDS) guidelines for EV-based therapeutic products (issued in December 2018) and followed the current good manufacturing practice standards for quality, nonclinical, and clinical assurance.

### Characterization of EVs

In accordance with the guidelines established by the International Society for Extracellular Vesicles (MISEV 2018) and regulatory standards issued by the Korean MFDS, EVs isolated from WJ-MSC-conditioned medium were thoroughly assessed for their physical morphology, particle size distribution, surface protein expression, sample purity, functional potency, therapeutic efficacy, long-term stability, and product safety [19].

### Bioinformatics

To identify the key therapeutic mediators within the EVs, microRNA (miRNA)-target networks were analyzed using normalized expression profiles from small RNA sequencing. Target genes of the total detected miRNAs were prioritized based on their cumulative regulatory weight, calculated by summing the linearized abundances of all targeting miRNAs. In contrast, for the abundant miRNAs, targets were identified and ranked according to the normalized expression levels of each individual miRNA. Validated target genes were retrieved using the multiMiR R package by exclusively querying human-specific interactions from integrated validated databases (miRecords, miRTarBase, and TarBase). The identified target genes were mapped to Entrez IDs and annotated with Gene Ontology (GO) biological process terms using AnnotationDbi, org.Hs.eg.db and GO.db packages. Subsequently, a custom rule-based classification function was applied to categorize the GO terms into 10 AD-relevant functional categories: pro-/anti-/regulatory angiogenesis, pro-/anti-/regulatory inflammatory, response to oxidative stress, keratinocyte biology, and Th1/Th2 and Th17 differentiation.

For proteomic analysis, a comprehensive list of proteins identified within the EVs was extracted. The gene symbols of these detected proteins were converted to Entrez IDs using the org.Hs.eg.db package, and Kyoto Encyclopedia of Genes and Genomes (KEGG) pathway enrichment analysis was performed using the enrich KEGG function of the clusterProfiler package to identify the functional pathways represented by the EV protein cargo. After excluding the pathways related to metabolic processes, disease-specific pathways, and those associated with gene expression regulation, the top 20 pathways were selected. All analyses were conducted in RStudio with the specified packages.

### Animal model of AD

Female BALB/c mice (6 weeks old, 18 to 20 g) were obtained from Orient Bio (Gapyeong, Republic of Korea). The mice were maintained under specific-pathogen-free conditions. The animals were randomly divided into 4 groups (*n* = 6 per group). The sample size was determined based on previous studies that demonstrated sufficient statistical power to detect differences in cytokine expression in similar AD models. To induce AD-like skin lesions, the shaved dorsal skin of the mice was treated with DFE (Citeq BV, Groningen, Netherlands) dissolved in PBS. Two days later, a 1% 1-chloro-2,4-dinitrobenzene (DNCB) solution (dissolved in acetone:olive oil = 3:1) was applied in a similar manner. After 3 weeks of repeated treatment, the mice were randomly divided into 5 groups: (a) healthy group (DFE–DNCB-untreated mice), (b) placebo group (PBS + DFE–DNCB-treated AD mice), (c) EV-treated group (a single subcutaneous injection of EVs [6 × 10^8^ particles/ml] in AD mice), (d) HP-treated group (the hydrogel was applied topically to the AD lesions once a day in AD mice), and (e) HP+EV-treated group (HP+EVs were applied topically to the AD lesions once a day in AD mice). The mice were sacrificed on day 35. After sacrifice, blood samples were collected by orbital puncture and the dorsal skin was collected for histological, immunohistochemical, and molecular analyses.

### Clinical evaluation of the severity of AD

The severity of skin lesions was evaluated 3 times per week, starting from 1 week post-initial induction. The dermatitis score was assessed by clinical visual evaluation, with symptoms scored from 0 to 3 (0, none; 1, mild; 2, moderate; and 3, severe). Skin lesion severity was reported as the total score for the following 4 symptoms: erythema, scarring/dryness, edema, and erosion [20].

### Immunohistochemistry

Ear and dorsal skin tissues were harvested from each mouse after euthanasia, fixed in 4% paraformaldehyde, and embedded in paraffin. The tissues were sectioned at a thickness of 5 μm and stained with a hematoxylin and eosin staining kit (ab245880; Abcam, Cambridge, UK) to evaluate epidermal thickness and inflammatory infiltration. To assess mast cell infiltration, skin sections were stained using the NovaUltra Toluidine Blue Stain Kit (IW-3013; IHC World, Ellicott City, MD, USA) according to the manufacturer’s instructions.

To evaluate the therapeutic effects of EVs on skin barrier integrity and oxidative stress, immunofluorescence staining was performed for filaggrin, occludin, and 8-hydroxy-2′-deoxyguanosine (8-OHdG). The dorsal skin tissues were fixed in 4% paraformaldehyde and blocked with 10% normal goat serum. The tissues were incubated overnight at 4 °C with goat anti-filaggrin antibodies (1:500; Abcam), anti-occludin antibodies (1:500; Cell Signaling Technology, Beverly, MA, USA), and anti 8-OHdG antibodies (1:500; Bioss, Woburn, MA, USA). The tissues were washed with PBS and incubated with secondary DyLight-labeled anti-goat immunoglobulin G (IgG) (1:200, 594 nm; Abcam) and anti-rabbit IgG antibodies (1:200, 488 nm; Vector Laboratories, Burlingame, CA, USA). Samples were imaged using a fluorescence microscope (EVOS; Advanced Microscopy Group, Bothell, WA, USA), and the relative fluorescence intensities were quantified using the ImageJ software (National Institutes of Health, Bethesda, MD, USA).

### Enzyme-linked immunosorbent assay

Approximately 1 g of dorsal skin tissue (*n* = 6) was homogenized in the presence of protease inhibitors, and protein extraction was performed using radioimmunoprecipitation assay buffer. The resulting homogenate was centrifuged at 12,000 × *g* for 30 min at 4 °C, after which the supernatant was carefully collected to avoid disturbing the pellet. The total protein concentration was quantified using the Bradford assay. The levels of inflammatory cytokines (interleukin-4 [IL-4], IL-13, and tumor necrosis factor-alpha [TNF-α]) in the supernatant were determined using enzyme-linked immunosorbent assay kits (BD Biosciences, Franklin Lakes, NJ, USA) according to the manufacturer’s guidelines. The absorbance was measured at 450 nm using a microplate reader (Molecular Devices, Downingtown, PA, USA).

### Western blot

Equal amounts of protein (20 μg) from EV samples were subjected to sodium dodecyl sulfate–polyacrylamide gel electrophoresis. Proteins were transferred onto nitrocellulose membranes, washed with 1× tris-buffered saline containing 0.1% Tween-20, and blocked with skim milk at room temperature (25 °C) for 1 h. The membranes were incubated overnight at 4 °C with primary antibodies against Alix, syntenin-1, cluster of differentiation 81 (CD81), CD9, and CD63 (Cell Signaling Technology, Danvers, MA, USA) (each at 1:1,000 dilution). The following day, horseradish peroxidase-conjugated anti-rabbit or anti-mouse IgG secondary antibodies were added for 1 h at room temperature (25 °C). Protein bands were detected using Enhanced Chemiluminescence Prime Western blotting Detection Reagent (GE Healthcare, Piscataway, NJ, USA), and images were acquired using the Amersham Imager 600 system (GE Healthcare Life Sciences).

### Quantitative RT-PCR

RNA acquisition, complementary DNA (cDNA) synthesis, and real-time polymerase chain reaction (RT-PCR) were performed as described previously [21,22]. The relative expression levels of the target messenger RNAs were normalized to those of glyceraldehyde 3-phosphate dehydrogenase (GAPDH) as the housekeeping gene. Total RNA was extracted from each experimental condition using the Hybrid-R reagent (GeneAll, Seoul, Republic of Korea). For reverse transcription, 500 ng of total RNA was converted into cDNA using oligo(dT) primers, with incubation performed at 55 °C for 60 min, followed by enzyme inactivation at 85 °C for 5 min. The resulting cDNA was stored at 4 °C until further analysis.

Quantitative RT-PCR (RT-qPCR) was carried out using Universal SYBR Green Master Mix (Applied Biosystems, Foster City, CA, USA), and amplification was performed with the following thermal cycling protocol: initial denaturation at 95 °C for 15 min, followed by 40 cycles of 95 °C for 30 s, 59 °C for 30 s, and 72 °C for 30 s. RT-qPCR was conducted by the StepOne RT-qPCR System (Applied Biosystems). Gene expression levels were calculated using the 2^−ΔΔCt^ method, comparing each target gene to GAPDH. The primer sequences used for RT-qPCR are listed in Table S1.

### Synthesis of HP

The HP hydrogel was synthesized by mixing HA sodium salt (30 to 50 kDa MWCO; Glentham Life Sciences, Corsham, UK) and PF127 (Sigma-Aldrich, St. Louis, MO, USA) in PBS for EV loading. To prepare the 30% PF127/1% HA hydrogel (HP30), PF127 powder was dissolved in a 1% (w/v) HA solution using a rotator at 4 °C for 24 h. Subsequently, the HP30 hydrogel was diluted with a 1% HA solution to obtain 25%, 19%, 17%, and 16% hydrogels (HP25, HP19, HP17, and HP16, respectively). All hydrogels were sterilized using a 0.22-μm polyethersulfone membrane syringe filter before use in experiments.

### Vial tilting

After preparing HP hydrogels at various concentrations, 500 μl of each hydrogel was aliquoted into vials. The samples were then equilibrated for 5 min at different temperatures: 4 °C (refrigeration), 28 °C (slightly above room temperature), and 37 °C (body temperature). The vials were subsequently inverted to observe the gelation behavior of each hydrogel.

### Rheological characterization

The rheological properties of the HP hydrogels were characterized using Discovery Hybrid Rheometer-1 (TA Instruments, New Castle, DE, USA). For each analysis, a hydrogel sample (200 μl) was loaded into the rheometer chamber. To determine the gelation temperature and temperature-dependent viscoelastic behavior, a temperature sweep was performed from 5 °C at a heating rate of 1.5 °C/min, with a fixed frequency of 1 Hz and 1% strain. In addition, shear-thinning behavior was evaluated by measuring the viscosity as a function of shear rate, which was gradually increased from 0.1 to 1,000 s^−1^ at a constant temperature of 37 °C. The storage modulus (*G*′), loss modulus (*G*′′), and viscosity changes were recorded for all hydrogel formulations.

### Degradation test

After preparing HP hydrogels at various concentrations, the degradation time was evaluated under PBS and hyaluronidase (HAase) (enzymatic conditions). Each hydrogel sample (100 μl) was placed in a 1.5-ml tube and fully immersed in 100 μl of PBS (Cytiva, Marlborough, MA, USA) and 100 μl of 100 U/ml HAase (Sigma-Aldrich, St. Louis, MO, USA). The tubes were incubated at 37 °C in a shaker for 48 h. The hydrogel-containing tubes were collected and washed 3 times with distilled water at predetermined time points (1, 2, 4, 8, 24, and 48 h). After washing, the samples were frozen at −80 °C for 24 h and subsequently lyophilized to measure the remaining weight and calculate the degradation ratio.

$$\text{Weight loss}\;\left(\%\right)=\frac{{\text{weight}}_{0}-{\text{weight}}_{t}}{{\text{weight}}_{0}}\times 100$$ (1)

weight_0_ is the dry weight of the hydrogel after lyophilization at 0 h; weight*_t_* is the dry weight of the hydrogel after lyophilization at time *t*.

### Liposome synthesis

Liposomes used in this experiment were synthesized by mixing 1,2-dimyristoyl-*sn*-glycero-3-phosphocholine (Avanti Polar Lipids, Alabaster, AL, USA) with cholesterol (Sigma-Aldrich) at a molar ratio of 2:1. The lipid mixture was dissolved in chloroform (Life Technologies) at a concentration of 10 mg/ml to prepare the lipid solution. The solvent was evaporated using a digital rotary evaporator (vertical type, Daihan Scientific, Republic of Korea) at 45 °C and 50 rpm until complete removal of chloroform, forming a thin lipid film. The resulting lipid film was rehydrated with 1 ml of distilled water to synthesize the liposomes. The synthesized liposomes were then extruded through Whatman Nuclepore polycarbonate membranes (0.2-μm pore size, GE Healthcare Life Sciences, Pittsburgh, PA) using a mini-extruder set (Avanti Polar Lipids) to obtain uniform vesicles. Liposomes prepared using this process were subsequently used in release experiments.

### Liposome releasing test

After preparing HP hydrogels at various concentrations, drug release behavior was evaluated using liposomes and Transwell polycarbonate membrane cell culture inserts (6.5-mm Transwell with 5.0-μm-pore polycarbonate membrane; Corning, Tewksbury, MA, USA). According to the manufacturer’s instructions, 100 μl of liposome-loaded HP was placed into each Transwell insert, and 600 μl of PBS was added to the lower well. The plates were incubated at 37 °C in a shaking incubator at 200 rpm, and the PBS in the lower wells was collected and replaced with fresh PBS at 1, 4, 8, 24, 48, and 72 h. The collected solutions were analyzed for liposome concentration using a qNano instrument (IZON Ltd., Christchurch, New Zealand) based on tunable resistive pulse-sensing technology, and the cumulative release was subsequently calculated.

### Swelling test

To evaluate the swelling ratio of HP hydrogels, 100 μl of each HP hydrogel formulation was carefully dispensed into a density kit. The initial weight was measured using a precision balance, and the samples were immersed in PBS for 48 h. After immersion, the final weight was recorded, and the swelling ratio was calculated.

$$\text{Swelling ratio}\;\left(\%\right)=\frac{{\text{weight}}_{t}-{\text{weight}}_{0}}{{\text{weight}}_{0}}\times 100$$ (2)

weight_0_ is the weight of the hydrogel at 0 h; weight*_t_* is the weight of the hydrogel at time *t*.

### Preparation of HP+EV

For each 100-μl preparation, 5 μl of EV suspension (4.29 × 10^8^ EVs/ml), 5 μl of 2% (w/v) HA solution, and 90 μl of PF127 dissolved in 1% (w/v) HA solution were combined under sterile conditions at 4 °C to maintain the liquid state of PF127. EVs and HA were premixed before the addition of PF127, and the final mixture was gently pipetted to ensure homogeneity without generating bubbles. The prepared HP+EV was aliquoted (100 μl) and stored at 4 °C until use.

### Skin permeation studies

In vivo skin permeation of 1,1′-dioctadecyl-3,3,3′,3′-tetramethylindocarbocyanine perchlorate (DiI)-labeled EVs was assessed on the dorsal skin of mice using confocal laser scanning microscopy (CLSM). The mice were anesthetized via inhalation of isoflurane (Hana Pharm Co., Ltd., Seoul, Republic of Korea). Dorsal hair was shaved and gently rinsed with a sterile saline solution. After a 48-h recovery period for skin barrier regeneration, 3 formulations were applied to separate 4-cm^2^ areas on the hairless dorsal skin of the same animal using direct topical pipetting:

• group A: 0.5 ml of DiI-labeled HP
• group B: 0.5 ml of hydrogel containing DiI-labeled HP-EVs
• group C: 0.5 ml of hydrogel containing DiI-labeled HP-EVs pretreated with proteinase K (PK) from Promega (Madison, WI, USA)

PK treatment was performed by incubating EVs with 20 μg/ml PK at 37 °C for 1 h, followed by the addition of a protease inhibitor cocktail to stop the reaction before hydrogel incorporation.

At 1, 2, 4, and 8 h postapplication, 4 mice from each group were sacrificed for sample collection. Skin tissues were cleaned, rapidly frozen in liquid nitrogen, embedded in optimal cutting temperature compound, and cryosectioned into 5- to 10-μm sections using a cryostat microtome (HM525NX, Thermo Fisher Scientific, Waltham, MA, USA). CLSM imaging was conducted to visualize and evaluate the EV penetration and distribution.

In a separate group, AD was induced in the dorsal skin before treatment. The same 3 formulations were applied to lesional skin areas by topical pipetting, and skin collection, processing, and CLSM analyses were performed at identical time points.

### Thiobarbituric acid reactive substance assay

Lipid peroxidation levels in the dorsal skin tissues of mice with DFE-/DNCB-induced AD were assessed using the TBARS Assay Kit (BO-TBR-200, BIOMAX, Republic of Korea), following the manufacturer’s instructions.

### Statistical analysis

Statistical analysis of the data was performed using the GraphPad Prism 8 software package (GraphPad Software, San Diego, CA, USA). The analysis of variance test was conducted to analyze all data. Values less than 0.05 were considered statistically significant. All data are presented as mean ± standard deviation (SD) or mean ± standard error of the mean. Bioinformatics analyses were performed using R (version 4.5.2) in RStudio. For proteomic data, gene symbols were averaged by gene and converted to Entrez IDs using org.Hs.eg.db (v3.22.0). KEGG pathway enrichment analysis was conducted with clusterProfiler via the enrichKEGG function. For miRNA analysis, miRNAs matched target genes. GO biological process terms were retrieved using org.Hs.eg.db and GO.db (v3.22.0). Chord diagrams were generated using OriginPro 2024 (SR1 10.1.0.178). All other analyses used default parameters unless specified.

## Results

### Optimization of the hydrogel formulation for topical application

**Fig. 2.**
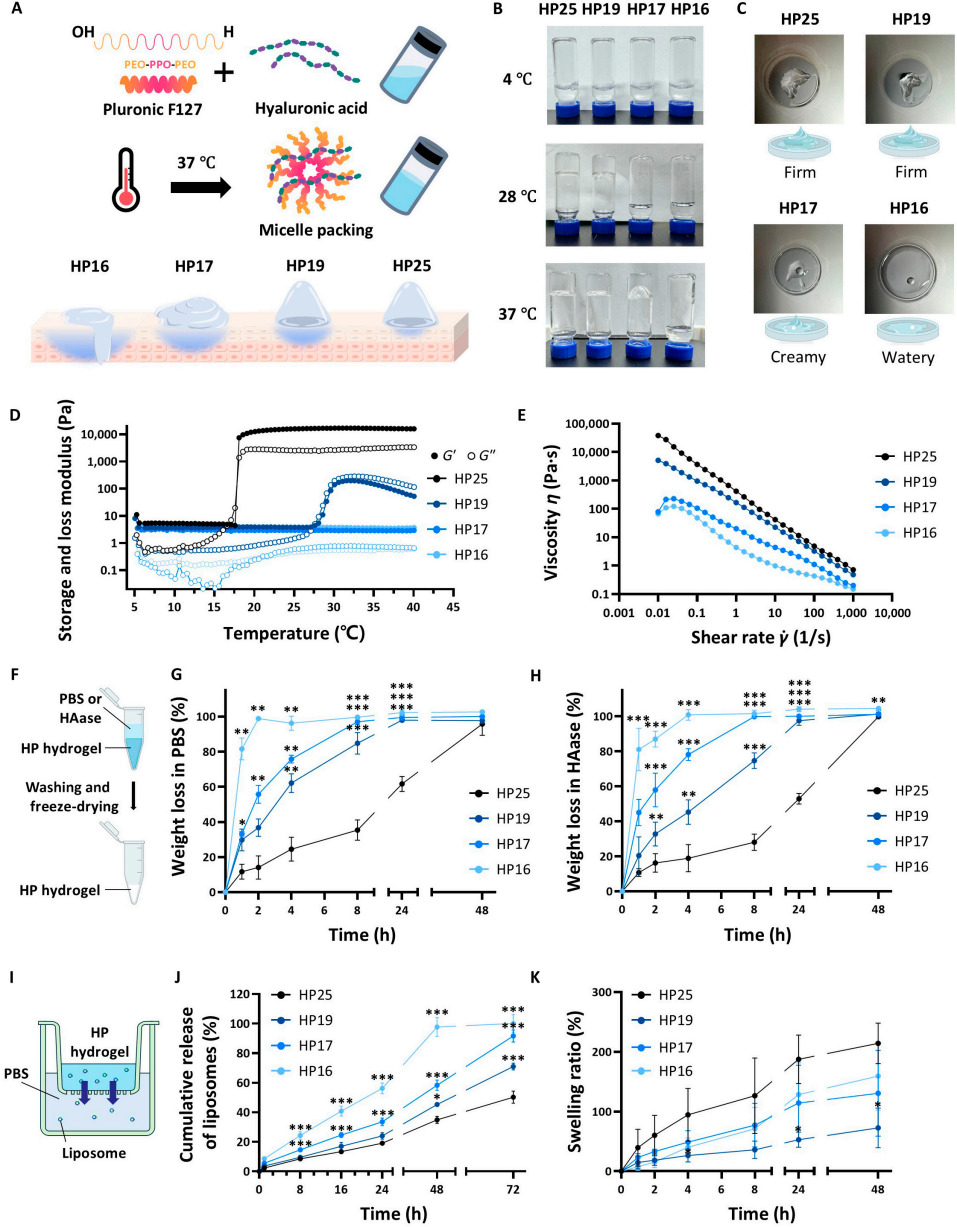
Optimization of hydrogel formulation for topical application of mesenchymal-stem-cell-derived extracellular vesicles (MSC-EVs). (A) Schematic illustration of the temperature-sensitive gelation process and behavior of hyaluronic acid/Pluronic F127 (HP) hydrogels at different concentrations. (B) Photographs of HP hydrogels prepared at different concentrations. (C) Photographs of the gels after a shear rate sweep test using a rheometer at 37 °C. (D and E) Rheological behavior of HP hydrogels. (F) Schematic diagram illustrating the HP hydrogel degradation assessment protocol. (G and H) Degradation profiles of hydrogels under phosphate-buffered saline (PBS) and hyaluronidase (HAase) conditions, respectively. (I) Schematic illustration of the experimental setup for evaluating liposome release from HP hydrogels using a Transwell plate. (J) Liposome release profiles of HP hydrogels at varying concentrations. (K) Swelling ratio of HP hydrogels as a function of PF127 concentration. Data are presented as mean ± standard deviation (SD), *n* = 3, and *P* values were calculated using one-way analysis of variance (ANOVA). *P < 0.05, **P < 0.01, and ***P < 0.001 compared with HP25.

We characterized the physicochemical properties of HP hydrogels to develop an optimal topical vehicle for EV delivery (Fig. 2A). PF127 is a nonionic triblock copolymer (poly(ethylene oxide)-poly(propylene oxide)-poly(ethylene oxide); PEO-PPO-PEO) that undergoes temperature-induced self-assembly; as the temperature increases, the hydrophobic PPO blocks dehydrate and aggregate into micellar cores, packing closely to form a physical gel [23]. We incorporated HA into this network to overcome the inherent limitations of pristine PF127, such as its rapid erosion or disintegration under physiological conditions. HA provides mechanical reinforcement and, crucially, maintains a moist environment that is favorable for skin hydration and barrier repair [24,25].

We optimized the PF127 concentration to achieve a formulation that allowed homogeneous EV loading at room temperature and optimal retention with uniform spreadability at body temperature. Visual observation of the gelation behavior (Fig. 2B and C) revealed distinct conditions of phase transition at different temperatures. Under refrigeration (4 °C), all candidates from HP16 to HP25 remained clear, low-viscosity sols that were ready for handling and preparation. When brought to a temperature slightly higher than room temperature (28 °C), the groups with higher concentrations, HP25 and HP19, had mostly transitioned to a gel state. This premature gelation is undesirable as it hampers the homogeneous mixing of EV suspensions. In contrast, HP17 and HP16 remained in the liquid–sol state, satisfying the critical requirement of uniform drug loading without physical stress or aggregation. Upon application at body temperature (37 °C), HP16 still remained in a sol state, leading to rapid runoff. In contrast, HP19 and HP25 formed rigid gels that were difficult to spread evenly. HP17, however, transitioned into a spreadable soft gel state with moderate viscosity while retaining its shape sufficiently to avoid runoff.

Rheological analysis further validated these observations (Fig. 2D and E). HP17 exhibited ideal shear-thinning behavior, where the viscosity decreased under the application of shear stress, allowing uniform spreading over sensitive AD lesions, and could be recovered immediately to form a stable depot of EVs. The degradation behavior of HP hydrogels was assessed in PBS and HAase environments within microcentrifuge tubes (Fig. 2F). All formulations exhibited time-dependent degradation, with complete degradation for most groups observed by 48 h (Fig. 2G and H). HP16 degraded almost completely within 4 h under both conditions. This excessively fast disintegration is largely attributed to its weak network, which is highly susceptible to fluidic shear-induced erosion during the mandatory washing steps. Conversely, HP25 exhibited the slowest degradation, retaining approximately 40% to 45% of its weight at 24 h with measurable traces up to 48 h. No significant differences were observed between PBS and HAase conditions.

Additionally, in vitro release profiles were evaluated within a structurally confined Transwell system using liposomes custom-fabricated by mimicking EVs’ physical properties (Fig. 2I and J and Fig. S1). Unlike the direct fluidic exposure in the degradation assay, this setup physically shields the hydrogel while maintaining a continuous sink condition through medium replacement, reflecting in vivo clearance. Under these conditions, the results showed that HP17 supported sustained release (>90% over 72 h), avoiding the rapid burst occurring in HP16 or the entrapment observed in HP25.

The swelling behavior of the HP hydrogels was closely correlated with their rheological properties and polymer network structure. At 37 °C, HP25 exhibited the highest swelling ratio. The opposite trend was observed at lower concentrations, where the swelling ratio increased as the concentration decreased from HP19 to HP16 (Fig. 2K). This behavior was attributed to the structural integrity of the gel network. Rheological analysis confirmed that only HP25 formed a complete gel (*G*′ > *G*′′) at about 18 °C with a robust network capable of maintaining structure during water absorption. In contrast, HP19 exhibited the lowest swelling. This behavior could be attributed to its delayed phase transition. As shown in the rheological profile, HP19 maintained a viscous-dominant state (*G*′ < *G*′′) even beyond 28 °C. Because of this delayed and incomplete micelle packing, the resulting network at 37 °C might lack the structural integrity required to restrict dissolution and support stable expansion. However, as the polymer concentration decreased further, the increased pore size and network flexibility might facilitate water diffusion, leading to the recovery of the swelling ratio. Consequently, HP17 was selected as the optimal carrier in this study, offering an ideal balance among mixing efficiency at room temperature (25 °C), uniform spreadability, and retention capability at body temperature.

### Characterization and molecular cargo analysis of clinical-grade MSC-EVs

**Fig. 3.**
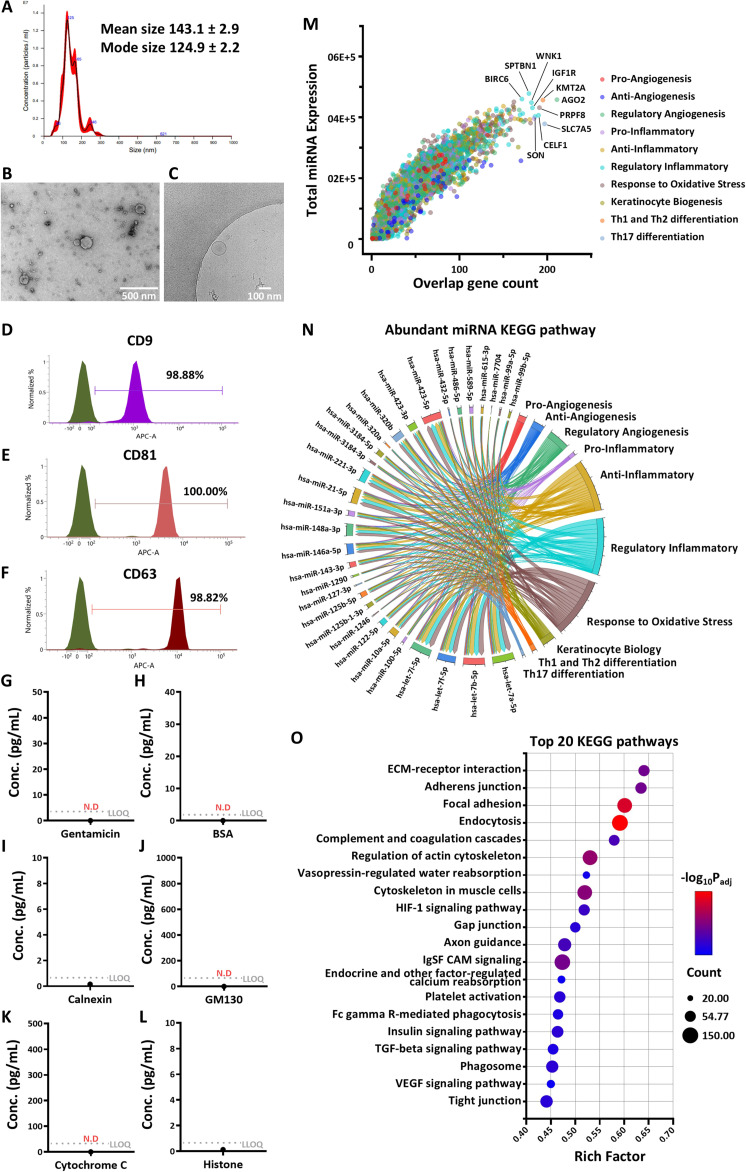
Characterization and molecular cargo analysis of clinical-grade mesenchymal-stem-cell-derived extracellular vesicles (MSC-EVs). (A) Size distribution and concentration of MSC-EVs determined using nanoparticle tracking analysis (NTA), presented as a histogram with mean and mode sizes indicated. (B and C) Morphology and ultrastructure of EVs visualized using transmission electron microscopy (TEM; scale bar = 500 nm) and cryo-electron microscopy (cryo-EM; scale bar = 200 nm). (D to F) Surface expression of tetraspanins (CD9, CD81, and CD63) assessed using flow cytometry, shown as histograms with percentage positivity. (G to L) EV-specific purity was assessed by detecting negative protein markers, including gentamicin, albumin, calnexin, histone H3, GM130, and cytochrome C, using enzyme-linked immunosorbent assay (ELISA). (M) Scatter plot depicting the relationship between total microRNA (miRNA) expression (*y*-axis, linear scale) and overlap gene count (*x*-axis, number of targeting miRNAs per gene) from small RNA sequencing data; points are color coded by functional categories (proangiogenesis, antiangiogenesis, regulatory angiogenesis, pro-inflammatory, anti-inflammatory, regulatory inflammatory, response to oxidative stress, keratinocyte biogenesis, helper T cell 1 [Th1]/Th2 differentiation, and Th17 differentiation), with top 10 targeted genes labeled. (N) Chord diagram illustrating enrichment analysis of abundantly expressed miRNAs, linking miRNAs to significantly enriched Kyoto Encyclopedia of Genes and Genomes (KEGG) pathways among their target genes; pathways are grouped and color coded by functional categories. (O) Bubble plot of the top 20 enriched KEGG pathways from proteomic analysis; bubble size represents gene count, color indicates −log_10_(*P*_adj_) (*P* value adjusted using the Benjamini–Hochberg method), and the *x*-axis shows the rich factor.

The clinical-grade MSC-EVs (SNE-101) used in this study exhibited a typical spherical morphology with a mean diameter of approximately 124 nm (Fig. 3A to C). Flow cytometry confirmed the expression of characteristic tetraspanins (exosome markers; CD9, CD81, and CD63) and the absence of cellular contaminants (Fig. 3D to L). To elucidate the molecular mechanisms underlying the therapeutic potential of these EVs, we comprehensively profiled their molecular cargo. Small RNA sequencing revealed a highly enriched miRNA repertoire. The scatter plot (Fig. 3M) illustrates the relationship between total miRNA expression levels (*y*-axis) and the overlapping gene count (*x*-axis), derived from experimentally validated target genes (miRecords, miRTarBase, and TarBase) categorized into 10 AD-relevant functional groups. This count represents the number of miRNAs in our dataset capable of targeting each gene related to therapeutic capacity for AD. Genes positioned in the upper right quadrant indicate stronger targeting by EV miRNAs, suggesting potential regulatory hotspots. Among the top 10 highly targeted genes, several were prominently labeled based on their functional categories: regulatory angiogenesis genes including argonaute RISC catalytic component 2 (AGO2); Th1 and Th2 differentiation genes including histone-lysine *N*-methyltransferase 2A (KMT2A); regulatory inflammatory genes including spectrin beta, non-erythrocytic 1 (SPTBN1), WNK lysine deficient protein kinase 1 (WNK1), insulin-like growth factor 1 receptor (IGF1R), baculoviral IAP repeat containing 6 (BIRC6), CUGBP Elav-like family member 1 (CELF1) and SON DNA, and RNA binding protein (SON); response to oxidative stress genes including pre-mRNA processing factor 8 (PRPF8); and Th17 differentiation genes including solute carrier family 7 member 5 (SLC7A5). These genes exhibited high miRNA expressions and substantial overlap counts, implying that EV miRNAs may preferentially modulate pathways associated with vascular remodeling, immune response, and cellular stress. Further analysis of the 4,307 genes associated with AD revealed their distribution across key biological categories potentially influenced by EV miRNAs. This profiling highlights the multifaceted role of EV miRNAs in balancing pro-, anti-, and regulatory pathways, with notable enrichment in oxidative stress and regulatory inflammatory genes, which could contribute to therapeutic strategies for skin disorders. To identify the key miRNA regulators driving these functional effects, we mapped the most abundant miRNAs to their validated targets across the 10 AD-relevant categories (Fig. 3N). Notably, the let-7 family (let-7a-5p, let-7b-5p, let-7f-5p, and let-7i-5p) showed the highest cumulative target gene counts across these categories, suggesting a predominantly regulatory role in EVs. Other miRNAs with a well-documented involvement in AD pathogenesis, including miR-21-5p, miR-143-3p, miR-10a-5p, and miR-146a-5p, were also highly enriched. Quantitative analysis of target genes revealed the strongest enrichment in “response to oxidative stress” (16,090 genes), “regulatory inflammatory” (15,305 genes), and “keratinocyte biology” (3,606 genes), indicating that the EVs are primed to target multiple pathological axes of AD simultaneously.

In parallel, the KEGG pathway analysis of the EV proteome revealed a focused set of pathways critical for skin integrity. After excluding metabolic and general processing categories, the analysis highlighted “ECM–receptor interaction” (rich factor ≈ 0.64), “adherens junction” (≈0.63), and “focal adhesion” (≈0.60) as the most significantly enriched pathways (Fig. 3O). These pathways involve key proteins such as integrins and junctional molecules, which are essential for cell–matrix and cell–cell adhesion to cooperate in maintaining the epithelial barriers between keratinocyte adhesion molecules. Additionally, the proteome was enriched in signaling pathways such as transforming growth factor-beta signaling, known for its anti-inflammatory effects suppressing immunoglobulin E (IgE) and TNF-α, and vascular endothelial growth factor signaling, which is linked to vascular permeability and itch in chronic lesions. This specific cargo profile suggests that our EVs are naturally equipped to not only suppress immune overactivation via miRNAs but also physically interact with and restore the damaged skin barrier via surface proteins.

### Alleviation of AD symptoms by topical HP+EV treatment

**Fig. 4.**
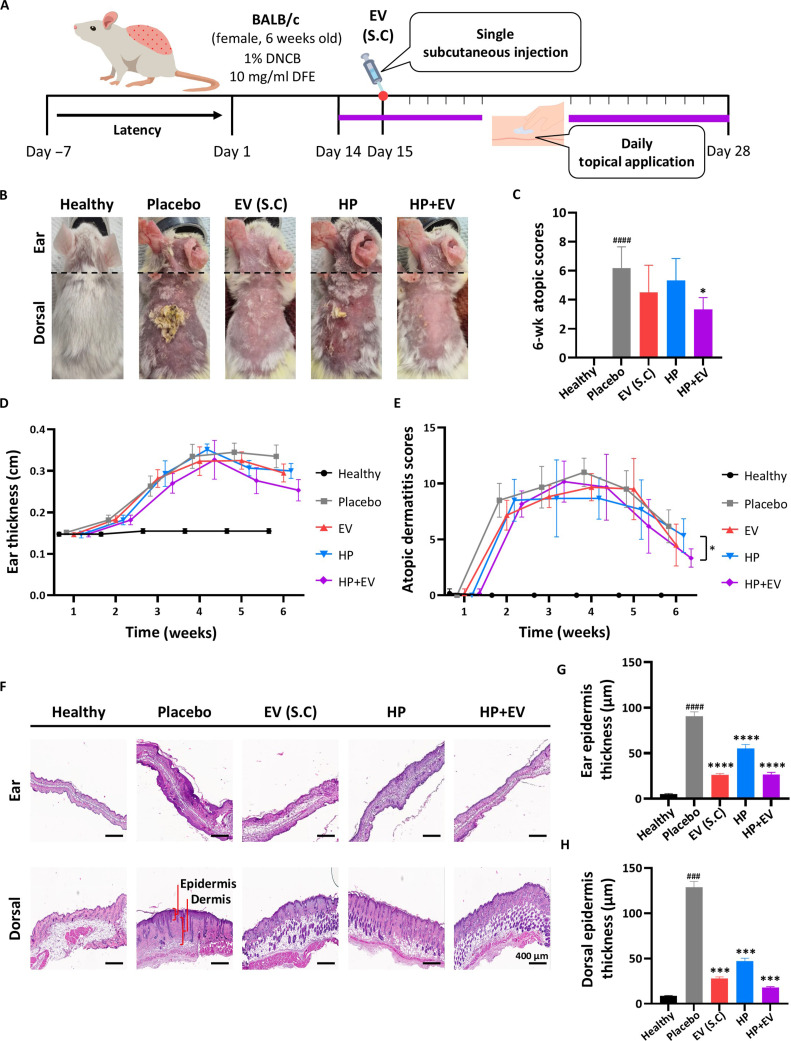
In vivo efficacy testing of atopic dermatitis (AD) symptoms after treatment with extracellular vesicles (EVs). (A) Design of the in vivo efficacy comparison study. (B) Photos of the ear and dorsal skin of mice in each group on day 35. (C) AD scores at week 6. (D and E) Ear thickness and AD scores for 6 weeks. (F) Representative hematoxylin- and eosin-stained ear and dorsal skin sections at week 6 (scale bar = 400 μm). (G and H) Epidermal thickness quantification in the ear and dorsal skin, respectively. Data are presented as mean ± standard error of the mean (SEM), *n* = 6, and *P* values were calculated using one-way analysis of variance (ANOVA). ^###^*P* < 0.001 and ^####^*P* < 0.0001 compared with the healthy group, and *P < 0.05, ***P < 0.001, and ****P < 0.0001 compared with the placebo group.

We evaluated the in vivo efficacy of the HP+EV formulation in a DFE-induced AD mouse model (Fig. 4A). As shown in Fig. 4B, the placebo group exhibited severe erythema, edema, and excoriation. However, topical application of HP+EV significantly ameliorated these clinical symptoms, reducing the dermatitis score to a level comparable to that of the positive control group treated with subcutaneous EV injection (EV (S.C)) (Fig. 4C to E). Histological analysis using hematoxylin and eosin staining confirmed that HP+EV treatment effectively reduced epidermal hyperplasia and dermal inflammation (Fig. 4F). Importantly, the reduction in ear and dorsal skin thickness in the HP+EV group was statistically different from that in the injection group, indicating that noninvasive topical delivery can achieve therapeutic potency equivalent to that of invasive administration (Fig. 4G and H).

### Broad immunomodulatory effects of HP+EVs on Th1/Th2-related cytokines

**Fig. 5.**
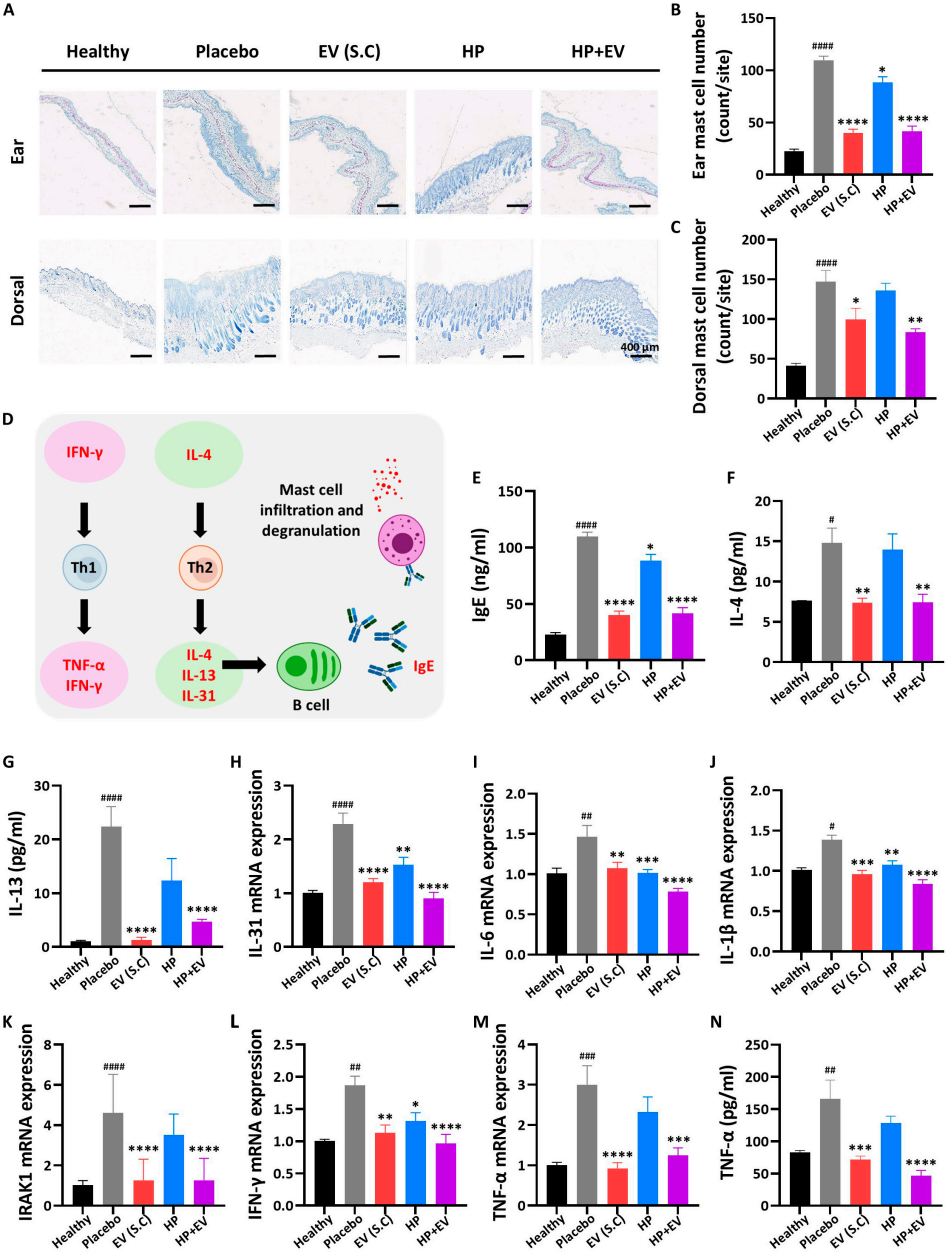
In vivo immunological analysis and messenger RNA (mRNA) expression of pro-inflammatory cytokines and Th1 and Th2 cytokine production in the dorsal skin of the atopic dermatitis (AD) mouse model. (A) Representative toluidine blue-stained sections of the ear and dorsal skin from each group showing mast cell distributions (purple metachromatic staining) (scale bar = 400 μm). (B and C) Quantification of mast cell number per area in the ear and dorsal skin. (D) Schematic representation of immune-responsive cytokine pathways in AD. (E) The production of immunoglobulin E (IgE) was detected using enzyme-linked immunosorbent assay (ELISA). (F to H) Helper T cell 2 (Th2) cytokines were estimated using real-time polymerase chain reaction (RT-PCR) analysis and ELISA. (I and J) Pro-inflammatory cytokines were quantified using RT-PCR analysis. (K to N) Th1 cytokines were analyzed using RT-PCR and ELISA. Data are presented as mean ± standard error of the mean (SEM), *n* = 6, and *P* values were calculated using one-way analysis of variance (ANOVA). ^#^*P* < 0.05, ^##^*P* < 0.01, ^###^*P* < 0.001, and ^####^*P* < 0.0001 are compared with the healthy group; *P < 0.05, **P < 0.01, ***P < 0.001, and ****P < 0.0001 are compared with the placebo group.

AD is driven by the complex interplay between Th2-dominant acute inflammation and Th1-associated chronic responses [26,27]. To investigate the molecular mechanism of immune regulation, we analyzed the cytokine profiles of skin lesions. Toluidine blue staining revealed that the number of mast cells was significantly reduced in the EV (S.C) and HP+EV groups compared to the placebo group (Fig. 5A to C). A schematic diagram illustrating the key immunological pathways involved in the pathogenesis of AD highlights the roles of Th cells, cytokines, and effector cells in driving inflammatory and allergic responses (Fig. 5D). On the left side, interferon-gamma (IFN-γ) stimulates the differentiation of Th1 cells, which in turn produce pro-inflammatory cytokines such as TNF-α and additional IFN-γ [28,29]. The Th1 pathway contributes to chronic inflammation and further tissue damage in AD [26,27]. In the central pathway, IL-4 promotes activation and differentiation of Th2 cells [28]. Th2 cells secrete IL-4, IL-13, and IL-31, which drive type 2 immune responses. IL-4 and IL-13 facilitate B cell activation and class switching, leading to IgE production [29]. IL-31 is associated with pruritus (itching), a hallmark of AD [30]. Upon allergen binding to IgE in mast cells, mast cell degranulation occurs, releasing histamine and other mediators that exacerbate itching, inflammation, and skin barrier dysfunction [31,32].

Consistent with the histological improvements, HP+EV treatment significantly down-regulated IgE and Th2-related cytokines, including IL-4 and IL-13, as well as the itch-associated cytokine IL-31 (Fig. 5E to H). Moreover, the treatment also effectively suppressed pro-inflammatory cytokines such as IL-6 and IL-1β, and Th1-related markers, such as interleukin-1 receptor-associated kinase 1 (IRAK1), IFN-γ, and TNF-α (Fig. 5I to N). This broad-spectrum suppression suggests that the cargo delivered by HP+EVs does not merely target a single pathway but also rebalances the overall immune microenvironment, effectively resolving both the acute and chronic phases of AD inflammation.

### Surface-protein-dependent skin penetration of EVs

Efficient delivery of therapeutics across the stratum corneum remains a critical challenge in topical AD treatments. To investigate the skin permeation kinetics of our platform, we monitored the distribution of DiI-labeled EVs in both healthy and AD-induced mouse skin.

**Fig. 6.**
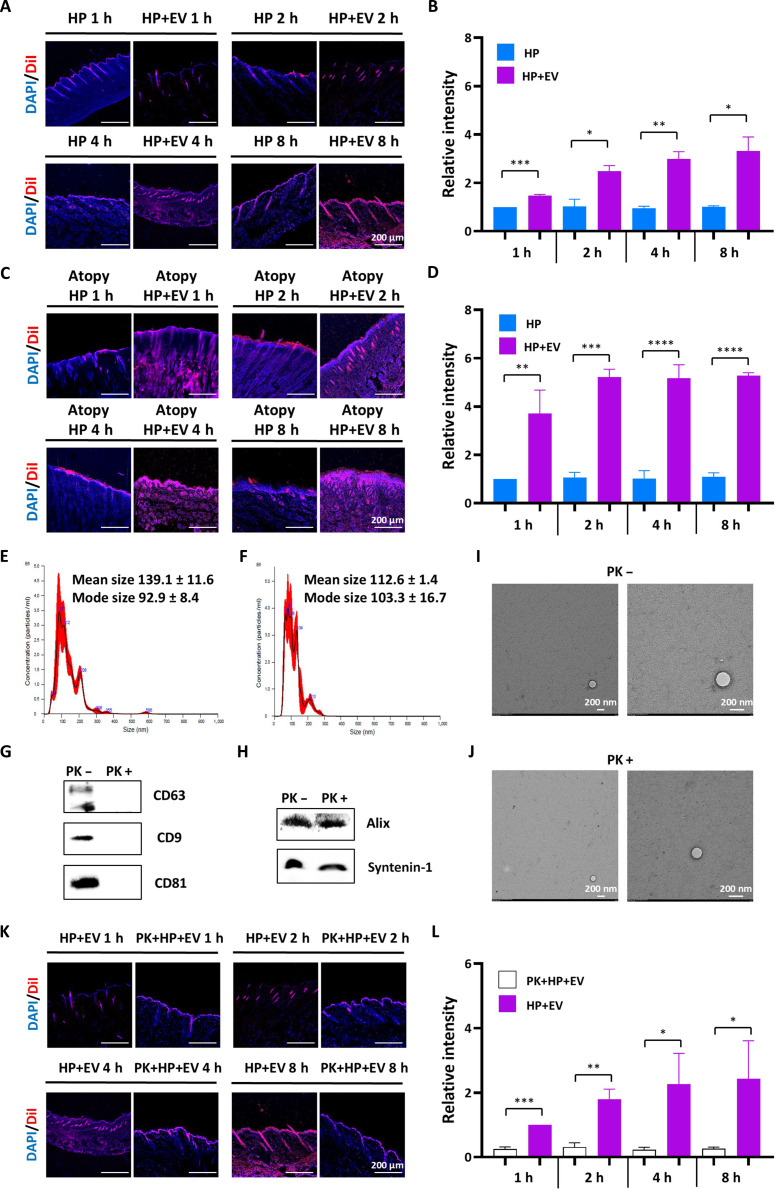
Mechanism of surface-protein-dependent skin penetration of mesenchymal-stem-cell-derived extracellular vesicles (MSC-EVs) through a compromised barrier. (A) Representative images showing the penetration of 1,1′-dioctadecyl-3,3,3′,3′-tetramethylindocarbocyanine perchlorate (DiI)-labeled EVs (red) in healthy mouse skin over time (1, 2, 4, and 8 h). Nuclei were counterstained with 4′,6-diamidino-2-phenylindole (DAPI; blue). Scale bar = 200 μm. (B) Quantitative analysis of the relative fluorescence intensity in healthy skin. (C) Representative images of DiI-labeled EV penetration in *Dermatophagoides farinae* extract (DFE)-induced atopic dermatitis (AD) mouse skin. (D) Quantitative analysis of the fluorescence intensity in AD-induced skin. (E and F) Nanoparticle tracking analysis (NTA) results of intact EVs and proteinase K (PK)-treated EVs, respectively, demonstrating that enzymatic shaving of surface proteins does not meaningfully alter the size distribution or concentration of the vesicles. (G and H) Western blot analysis of EV markers of surface tetraspanins and lumen, respectively, following PK treatment. (I and J) Transmission electron microscopy (TEM) images of intact EVs and PK-treated EVs, respectively. Scale bar = 200 nm. (K) Representative images comparing the skin penetration of intact hyaluronic acid/Pluronic F127-loaded EVs (HP+EVs) and PK-treated HP+EVs in AD lesions. (L) Quantitative analysis of the fluorescence intensity. Data are presented as mean ± standard error of the mean (SEM), *n* = 3. Statistical significance was determined by the Student *t* test: *P < 0.05, **P < 0.01, ***P < 0.001, and ****P < 0.0001.

In healthy skin with an intact barrier, fluorescence signals appeared to be confined to the uppermost layers of the stratum corneum, showing slow EV penetration rates during 8 h of application (Fig. 6A and B and Fig. S2). In contrast, EVs applied to AD-induced skin revealed a significantly enhanced permeation profile (Fig. 6C and D and Fig. S2). The compromised skin barrier in AD lesions facilitated rapid EV entry, with strong red fluorescence signals detected in the deeper dermis within just 2 h. Quantitative assessment demonstrated a time-dependent accumulation of EVs, showing a 3- to 5-fold increase in fluorescence intensity compared with that in the HP group. This suggests that the disrupted barrier in AD, paradoxically, serves as a portal for effective EV delivery.

To determine whether this enhanced penetration is a passive diffusion process or an active mechanism mediated by EV surface proteins, the surface proteins of EVs were enzymatically cleaved using PK. EV characterization results demonstrated that PK treatment only removed surface proteins without disrupting EV integrity. Nanoparticle tracking analysis results showed that PK-treated EVs maintained a size distribution similar to that of native EVs (Fig. 6E and F). Western blot analysis confirmed the complete removal of transmembrane tetraspanins (CD63, CD9, and CD81) (Fig. 6G), whereas luminal markers (Alix and syntenin-1) remained intact (Fig. 6H). Furthermore, transmission electron microscopy imaging verified that the vesicular morphology was preserved after PK treatment (Fig. 6I and J).

Intriguingly, the removal of surface proteins resulted in a dramatic loss of skin permeability. When applied to AD skin, the PK-treated EVs (PK+HP+EV) failed to penetrate the epidermis, showing fluorescence patterns mostly confined to the surface, similar to the vehicle control (HP) (Fig. 6K and Fig. S2). Quantitative analysis revealed that the fluorescence intensity of PK-treated EVs was significantly lower than that of intact EVs at all time points (Fig. 6L). These findings provide convincing evidence that EV skin permeation is not merely a result of passive diffusion through a broken barrier but is governed by surface-protein-dependent interactions with the skin matrix.

### Restoration of skin barrier and reduction of oxidative stress

**Fig. 7.**
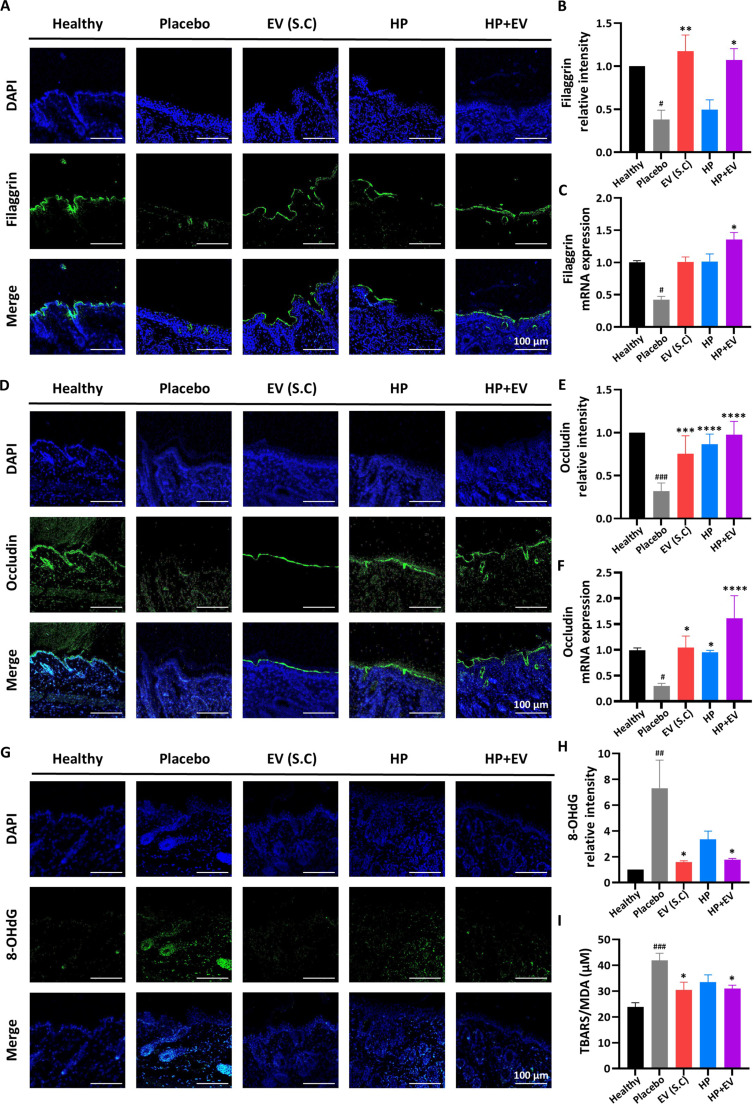
Restoration of skin barrier integrity and alleviation of oxidative stress by topical hyaluronic acid/Pluronic F127-loaded extracellular vesicle (HP+EV) treatment. (A) Representative immunofluorescence images of filaggrin (green) in dorsal skin tissues across groups. Scale bar = 100 μm. (B) Relative fluorescence intensity (*n* = 3). (C) Messenger RNA (mRNA) expression levels of filaggrin (*n* = 6). (D) Representative immunofluorescence images of occludin (green). Scale bar = 100 μm. (E) Relative fluorescence intensity (*n* = 3). (F) mRNA expression levels of occludin (*n* = 6). (G) Representative immunofluorescence images of 8-hydroxy-2′-deoxyguanosine (8-OHdG; green), scale bar = 100 μm, and (H) its quantitative analysis (*n* = 3). (I) Assessment of lipid peroxidation. Thiobarbituric acid reactive substance (TBARS)/malondialdehyde (MDA) levels in the dorsal skin (*n* = 6). Data are presented as mean ± standard error of the mean (SEM), and *P* values were calculated using one-way analysis of variance (ANOVA). ^#^*P* < 0.05, ^##^*P* < 0.01, and ^###^*P* < 0.001 are compared with the healthy group; *P < 0.05, **P < 0.01, ***P < 0.001, and ****P < 0.0001 are compared with the placebo group.

Finally, we examined whether the HP+EV treatment could repair the disrupted skin barrier in AD. Immunofluorescence staining revealed a robust restoration of filaggrin and occludin expression in the HP+EV-treated AD lesions, whereas the placebo and HP groups continued to show the diminished protein levels characteristic of impaired skin barriers (Fig. 7A to F). To elucidate whether the restoration of barrier proteins was correlated with the alleviation of oxidative stress, we evaluated representative ROS markers, including 8-OHdG and malondialdehyde. As illustrated in Fig. 7G and H, the levels of 8-OHdG, a hallmark of oxidative DNA damage, were markedly reduced in the HP+EV group compared with those in the placebo and HP groups. In addition, the level of malondialdehyde, a marker of lipid peroxidation, significantly decreased in the dorsal skin of the HP+EV group compared with those in the placebo and HP groups (Fig. 7I).

These findings, integrated with our EV cargo analysis, suggest that HP-delivered EVs directly supply antioxidant and barrier-restoring components to the lesion, effectively disrupting the deleterious cycle of barrier dysfunction and ROS-driven inflammation.

## Discussion and Conclusion

The present study established that a thermosensitive hydrogel-based topical delivery system (HP+EV) effectively overcomes the limitations of both injectable biologics and conventional topical formulations for AD treatment. Direct topical application of free EVs in simple buffers typically results in rapid clearance, desiccation, and loss of bioactivity. While previous hydrogel studies have primarily focused on the sol–gel transition for retention, our study highlights the importance of uniform spreadability in treating broad and irregular inflammatory lesions of AD. Because the compromised epidermal barrier in AD is highly vulnerable to mechanical stress, friction during topical application could possibly induce epidermal injury and trigger pro-inflammatory cytokine release. The robust shear-thinning capability of HP17 could mitigate this potential problem by ensuring uniform, friction-free spreadability over rough lesions [33]. Unlike liquid formulations that run off or stiff gels that cause application stress, our optimized vehicle forms a stable hydrated depot. This depot facilitates the sustained release of EVs into the stratum corneum, thereby maximizing the contact area and therapeutic timeframe. Furthermore, despite this prolonged retention, daily topical applications did not lead to matrix accumulation or skin occlusion in vivo. The physically cross-linked and thermoreversible properties of HP17 allow a rapid gel-to-sol transition upon contact with cool water, ensuring effortless washability during routine daily cleansing [34].

Using this patient-friendly platform, we demonstrated that topical HP+EV treatment exerts therapeutic effects comparable to those of direct subcutaneous injections. This is a notable finding, as it suggests that noninvasive delivery can replace painful injections without compromising efficacy. HP+EV treatment successfully reduced clinical AD scores, epidermal thickening, and mast cell infiltration. Notably, the HP+EV treatment exerted a broad-spectrum anti-inflammatory effect, effectively suppressing both Th2 cytokines (IL-4, IL-13, and IL-31), which drive acute-phase symptoms and pruritus, and Th1/pro-inflammatory cytokines (TNF-α and IFN-γ) implicated in disease chronicity and the atopic march [26,27].

The robust therapeutic efficacy observed in vivo was strongly supported by the specific molecular cargos of clinical-grade MSC-EVs. Our in vivo testing results showed substantial restoration of skin barrier proteins (filaggrin and occludin) and reduction in oxidative stress [35–38]. This aligns with our proteomic data, which revealed enrichment of proteins involved in extracellular matrix (ECM)–receptor interactions and adherens junctions. These EV-associated adhesion molecules may facilitate direct interactions with keratinocytes, promoting the structural repair of the physical barrier [39]. Simultaneously, the suppression of inflammation can be attributed to the abundance of immunomodulatory miRNAs, such as the let-7 family, miR-21, miR-143, and miR-146a, which broadly target AD-relevant pathways, as detected by our RNA sequencing analysis. Previous studies have reported that these miRNAs target key inflammatory signaling pathways, including nuclear factor-kappa B, thereby dampening the cytokine storm [6]. These findings, integrated with our EV cargo analysis, suggest that HP-delivered EVs directly supply antioxidant regulatory miRNAs (e.g., targeting the “response to oxidative stress” pathways) while simultaneously removing the primary sources of ROS by resolving the inflammatory cellular infiltrates. This dual mechanism effectively disrupts the deleterious cycle of barrier dysfunction and ROS-driven inflammation. Collectively, these results establish that the therapeutic outcomes, including skin barrier repair and immune modulation, are directly orchestrated by the distinct protein and RNA cargo encapsulated within our EV platform, rather than being secondary effects of the delivery vehicle.

Furthermore, we provide critical insights into the mechanism of transdermal delivery of EVs to AD lesions. Our data indicated that the compromised barrier in AD lesions acts as a “window of opportunity” for EV penetration. However, this penetration did not appear to be passive; the loss of permeation following PK treatment of the EV surface confirmed that surface proteins on EVs are necessary for their entry into the dermis. This aligns with previous reports that EV surface molecules determine tropism and uptake by skin-resident cells, such as keratinocytes and macrophages [40]. In addition, the incorporation of EVs into our HP hydrogels enhanced transdermal bioavailability by prolonging residence time, providing a hydrated microenvironment that stabilized vesicles, and promoted interaction with the stratum corneum. Embedding EVs in hydrogels has been shown to improve retention, enable controlled release, and enhance therapeutic outcomes compared to embedding EVs in solution [41].

Despite these promising results, this study has some limitations that require further investigation. Although DiI tracking of EVs and protease experiments suggested that EV surface proteins facilitate transdermal uptake, the precise molecular mechanisms governing skin penetration and cell-specific targeting remain to be elucidated. Similarly, while our comprehensive omics profiling revealed the pleiotropic effects of MSC-EVs on oxidative stress and barrier repair, the exact contribution of individual molecular drivers was not isolated in this study. Given the complex, combinatorial nature of the EV cargo and its functional redundancy, pinpointing a single downstream effector remains challenging. Future studies employing functional knockout models (e.g., small interfering RNA) for specific highly enriched miRNAs or proteins will be valuable to further elucidate the precise molecular hierarchy underlying the therapeutic efficacy. Additionally, although we demonstrated efficacy in a murine model, human skin has a thicker stratum corneum, which may affect penetration efficiency. Future studies should utilize ex vivo human skin models to validate transdermal delivery rates. Finally, as the present study utilized an acute AD model, future investigations using chronic AD models are needed to evaluate the multimodal efficacy shown in this study, as well as long-term safety, ensuring that repeated topical application does not induce tolerance or local irritation over extended periods.

In conclusion, this study establishes the HP+EV hydrogel as a clinically viable solution to a long-standing trade-off between the high potency of biologics and the practical convenience of topical treatments. We believe that this next-generation EV–hydrogel system could offer a robust foundation for the clinical translation of cell-free, noninvasive biotherapeutics for chronic inflammatory skin diseases, potentially improving patient compliance and quality of life.

## Data Availability

The data that support the findings of this study are available from the corresponding authors upon reasonable request.
