# Supplementary material for: Thermosensitive Hydrogel Enables Noninvasive Extracellular Vesicle Therapy for Atopic Dermatitis
Source: Biomater Res. 2026 May 18;30:0368. doi: 10.34133/bmr.0368 (PMC13181167; doi:10.34133/bmr.0368)
Supplement: Supplementary 1 — Figs. S1 and S2 Table S1 [file bmr.0368.f1.docx]

SUPPLEMENTARY MATERIALS


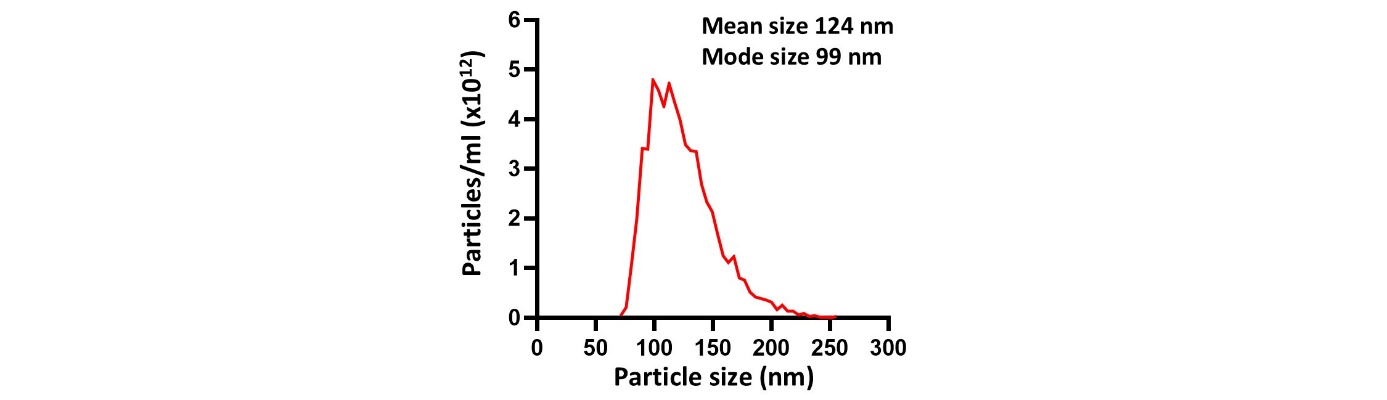


**Figure S1.** Liposome size distribution and concentration

The liposome size distribution and concentration were determined using TRPS technology and presented as histograms. The liposomes exhibited a unimodal distribution with a modal particle diameter of 99 nm and a mean diameter of 124 nm, indicating a relatively homogeneous population with moderate polydispersity.


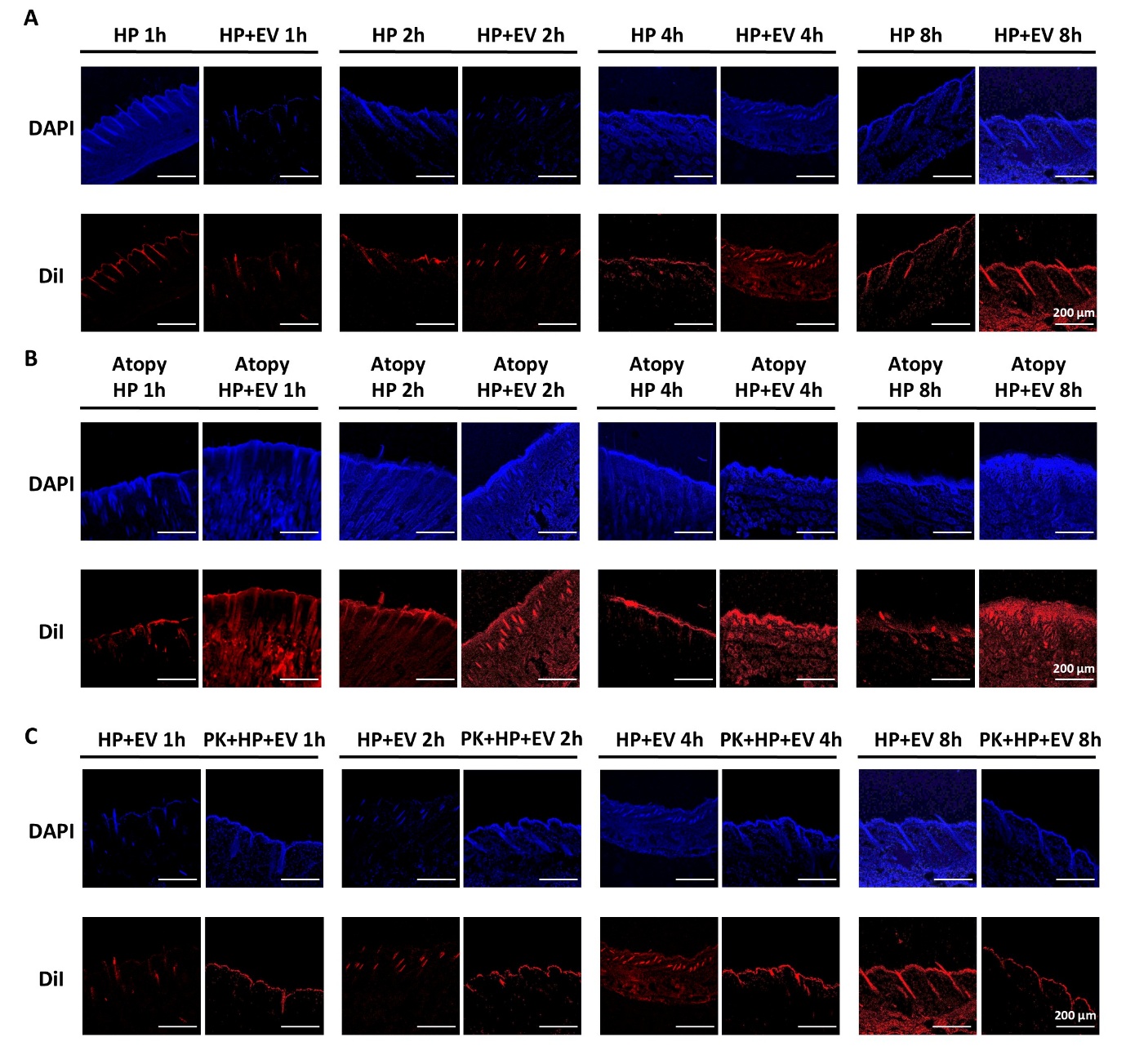


**Figure S2.** Evaluation of skin permeability of DiI-labeled EVs topically applied with HP

(A) Time-course fluorescence images of skin sections from healthy BALB/c mice after topical application of Dil-labeled HP hydrogel alone (HP) or DiI-labeled EVs incorporated in hydrogel (HP+EV) for the indicated durations (1, 2, 4, and 8 h). Upper row: DAPI (nuclei, blue); lower row: DiI (HP, EVs, red). (scale bar = 200 µm). (B) Time-course fluorescence images of skin sections from DNCB/DFE-induced AD BALB/c mice treated under the same conditions with HP or HP+EV for 1, 2, 4, and 8 h. Upper row: DAPI; lower row: DiI. (scale bar = 200 µm). (C) Time-course fluorescence images of skin sections from AD BALB/c mice after topical application of DiI-labeled untreated EVs (HP+EV) or PK-treated EVs (PK+HP+EV) incorporated in hydrogel for the indicated durations (1, 2, 4, and 8 h). Upper row: DAPI; lower row: DiI. (scale bar = 200 µm).

**Table S1.** List of primers used in real‐time PCR analysis

| **Gene** | **Forward Primer sequence (5ʹ→3ʹ)** | **Reverse Primer sequence (5ʹ →3ʹ)** |
| --- | --- | --- |
| IL-31 | (F) 5ʹ-CACACAGGAACAACGAAGCC-3ʹ | (R) 5ʹ-CGATATTGGGGCACCGAAG-3ʹ |
| IL-1β | (F) 5ʹ-TCAGCCAATCTTCATTGCTCAA-3ʹ | (R) 5ʹ-TGGCGAGCTCAGGTACTTCTG-3ʹ |
| IL-6 | (F) 5ʹ-AGGGCTCTTCGGCAAATGTA-3ʹ | (R) 5ʹ-GAAGGAATGCCCATTAACAACAA-3ʹ |
| TNF-α | (F) 5’-GCAGGTCTACTTTGGGTCATTG-3’ | (R) 5’-GCGTTTGGGAAGGTTGGA-3’ |
| IFN-γ | (F) 5ʹ-CTGATGGGAGGAGATGCTA-3ʹ | (R) 5ʹ-CGGGTGTAGTCACAGTTTTC-3ʹ |
| Occludin | (F) 5ʹ-TGGCAAGCGATCATACCCAGAG-3ʹ | (R) 5ʹ-CTGCCTGAAGTCATCCACACTC-3ʹ |
| Filaggrin | (F) 5ʹ-ATGTCCGCTCTCCTGGAAAG-3ʹ | (R) 5ʹ-TGGATTCTTCAAGACTGCCTGTA-3ʹ |
| GAPDH | (F) 5ʹ-GCCACATCGCTCAGACACC-3ʹ | (R) 5ʹ-CCCAATACGACCAAATCCGT-3ʹ |
| IRAK1 | (F) 5ʹ-TGTGGACACCGATACCTTCA-3ʹ | (R) 5ʹ-GCTTTTCAGGGTCACTCCAG-3ʹ |
